# A Pre-Adoption Assessment of a Collaborative Care Approach to Dental-Fear Treatment

**DOI:** 10.3390/oral4040046

**Published:** 2024-12-06

**Authors:** Kelly A. Daly, Richard E. Heyman, Alison L. Drew, Amy M. Smith Slep, Rochelle Bubis, Jahyung (Jai) Lee, Victoria V. V. Pearce, Racquel Jones, Marissa Ruggiero, Mark S. Wolff

**Affiliations:** 1 College of Dentistry, New York University, New York, NY 10010, USA;; 2 School of Dental Medicine, University of Pennsylvania, Philadelphia, PA 19104, USA;

**Keywords:** behavioral health, dental fear, interprofessional collaboration, access to care

## Abstract

**Background:**

With an estimated global prevalence rate of over 30%, dental fear is a ubiquitous public health phenomenon. Dental fear’s adverse effects on patient oral health and quality of life are well established; the stresses and financial repercussions it can pose to providers are increasingly recognized. Although dental fear is highly treatable, a disseminable intervention that satisfies the needs of patients and dental care providers has yet to be realized.

**Objectives:**

We sought to understand allied dental professionals’ experiences treating patients with dental fear and their impressions of, and receptivity to, a stepped-care approach, including perceived barriers and facilitators to its adoption.

**Methods:**

Thirteen semi-structured focus groups comprising students in (a) current dental hygiene programs and (b) other dental programs, who had prior experience working as licensed dental hygienists or allied professionals (i.e., dental assistants, dental therapists, dental treatment coordinators; *N* = 49) were conducted. Focus group discussions were recorded, transcribed, and thematically analyzed.

**Results:**

Participant responses supported the need for innovation in managing dental fear in practices, and supported hygienists as likely facilitators of any new behavioral approach. Overall, participants were receptive to the idea of a stepped-care treatment approach but identified several factors (e.g., accessibility, costs, treatment credibility) that would need to be addressed for its adoption by patients and dental professionals.

**Conclusion:**

A stepped-care approach to dental fear treatment has promise to meet the needs of patients and dental professionals. Potential barriers and facilitators to adoption provide a roadmap for future intervention refinement and efficacy testing.

## 1. Introduction

U.S. and global studies of dental fear report a prevalence of over 30% for adults [1]. Although individuals with moderate-to-severe dental fear are more likely to avoid preventive care [2] they nevertheless constitute over 20% of U.S. adult [3,4] and child [5,6] dental patients. Fear and subsequent avoidance of prophylactic care often led to more acute dental contacts that involve pain—thus punishing attendance (reinforcing continued avoidance) and resulting in a vicious cycle of fear, avoidance, and negative dental experiences [7]. Over time, this culminates in worse oral and systemic health and diminished quality of life [8].

Tackling dental fear at scale (e.g., in the United States alone this equates to 78 million adults, 36 million of whom see a dentist in a given year) necessitates a solution that meets the needs of both patients and dental professionals [9] which the current gold-standard psychological treatment does not. Comprising techniques to help individuals alter anxiety-driven cognitions, regulate emotional responses, and cope with feared stimuli, cognitive-behavioral treatment for dental fear (CBT-DF) has shown efficacy across nearly two dozen clinical trials [10]. Despite its effectiveness, CBT-DF has multiple limitations: It is rarely available outside of specialty clinics [11], its administration necessitates knowledge and skills outside the scope of most dental professionals [12], and it requires 3–6-h of in-person treatment—almost of all of which requires a dental chair—making it time and cost prohibitive in typical dental practices [13].

A stepped-care (progressive treatment dosing) approach to dental fear, as has been developed by this research group [14], may provide a more feasible treatment model. Leveraging technology, CBT-DF was adapted such that the first step of the treatment is self-administered through a mobile app on smartphones or tablets. The second step, reserved only for those who do not sufficiently benefit from the app, consists of a one-hour individually tailored in-chair treatment with a mental health provider.

Dental hygienists and other allied dental health professionals have crucial roles to play in the adoption and implementation of a stepped-care approach. Since its inception, the scope of dental hygiene has evolved from a sole focus on oral prophylactic treatment to holistic care that acknowledges connections between patients’ oral and general health, and overall well-being [15]. Hygienists’ training prioritizes interpersonal communication and education of patients [16], and often demands competence in motivational interviewing [17]. Patients spend considerably more time one-on-one with their hygienists, experience less fear of hygienists-facilitated treatment, and express more positive attitudes toward hygienists than dentists—even feeling more comfortable with hygienists administering anesthetics [18–20].

Hygienists have been at the forefront of behavioral health innovations in oral healthcare, including participating in multidisciplinary collaborations with mental healthcare providers [21,22] and providing brief interventions for tobacco cessation [23] and heavy alcohol use [24]. Moreover, dental hygienists are the targets of several programs to improve screening and referral for eating disorders [25], substance use disorders [26], and intimate partner violence [27].

Dental hygienists are in a unique position to screen and educate fearful patients, a role that suits their training and strengths [4,28]. Disseminable stepped-care approaches have the potential to improve the experiences of fearful patients and the dental staff who care for them. Using this approach, dental staff screen and refer patients to an accessible app-based intervention (step 1) and facilitate mental health providers’ provision of one-on-one cognitive-behavioral in-chair treatment to fearful patients (step 2). The researchers sought to understand the perceptions of hygienists and allied dental staff regarding current barriers to treating dental fear and whether a stepped-care intervention may prove an appropriate method for meeting the needs of patients and dental professionals.

The study aims were to elicit (a) hygienists’ and allied dental professionals’ experiences of identifying and treating patients with dental fear in practices and (b) their impressions, informed by clinical experience, of practices’ receptivity to a stepped-care approach for treating dental fear. Three research questions guided this study: (a) how have hygienists and allied dental health professionals observed practitioners assessing and addressing patients’ dental fear? (b) what are their impressions of the stepped-care approach and its feasibility for treating dental fear in the settings where they have trained? and (c) what factors are perceived to influence dental practices’ adoption of this type of care model?

## 2. Materials and Methods

### 2.1. Participants and Procedures

Participants (a) had experience working as an allied dental professional (i.e., dental hygienist, dental assistant, dental therapist, treatment coordinator) or (b) were current dental hygiene students (see Table 1 for participant demographics). Participants were recruited from two large dental colleges in the northeastern United States. An email was sent to all students, inviting those with prior or current experience working in private dental offices as hygienists or allied dental staff to participate in a focus group.

A total of 13 focus groups were conducted between August 2020 and March 2021. Each focus group was facilitated by either (a) Ph.D.-level psychologists or (b) dental-student research assistants. Initial focus groups were monitored by members of the research team to ensure adherence to the protocol. Before beginning the focus group, participants completed a consent form and a brief demographics questionnaire online via REDcap. Groups began with facilitators introducing themselves and their role in guiding the session. Participants were asked to introduce themselves briefly. A facilitator then read a brief script setting ground rules for the focus group and explaining its purpose. Participants were asked to focus their comments on the practice(s) where they had worked or shadowed, not on their classroom or within-school clinic experiences.

A semi-structured protocol, addressing the study’s overarching research questions, guided the discussion. First, facilitators asked participants to describe how the practices where they had worked identified and typically treated patients with dental fear. Next, a facilitator read a paragraph explaining the stepped-care approach—the novel inclusion of the self-guided dental fear app plus a single-hour one-on-one session with a mental health provider at the dental practice (if needed). Participants were asked for their feedback on perceived barriers and facilitators to adopting the stepped-care approach at the practices where they had worked.

**Figure 1.**
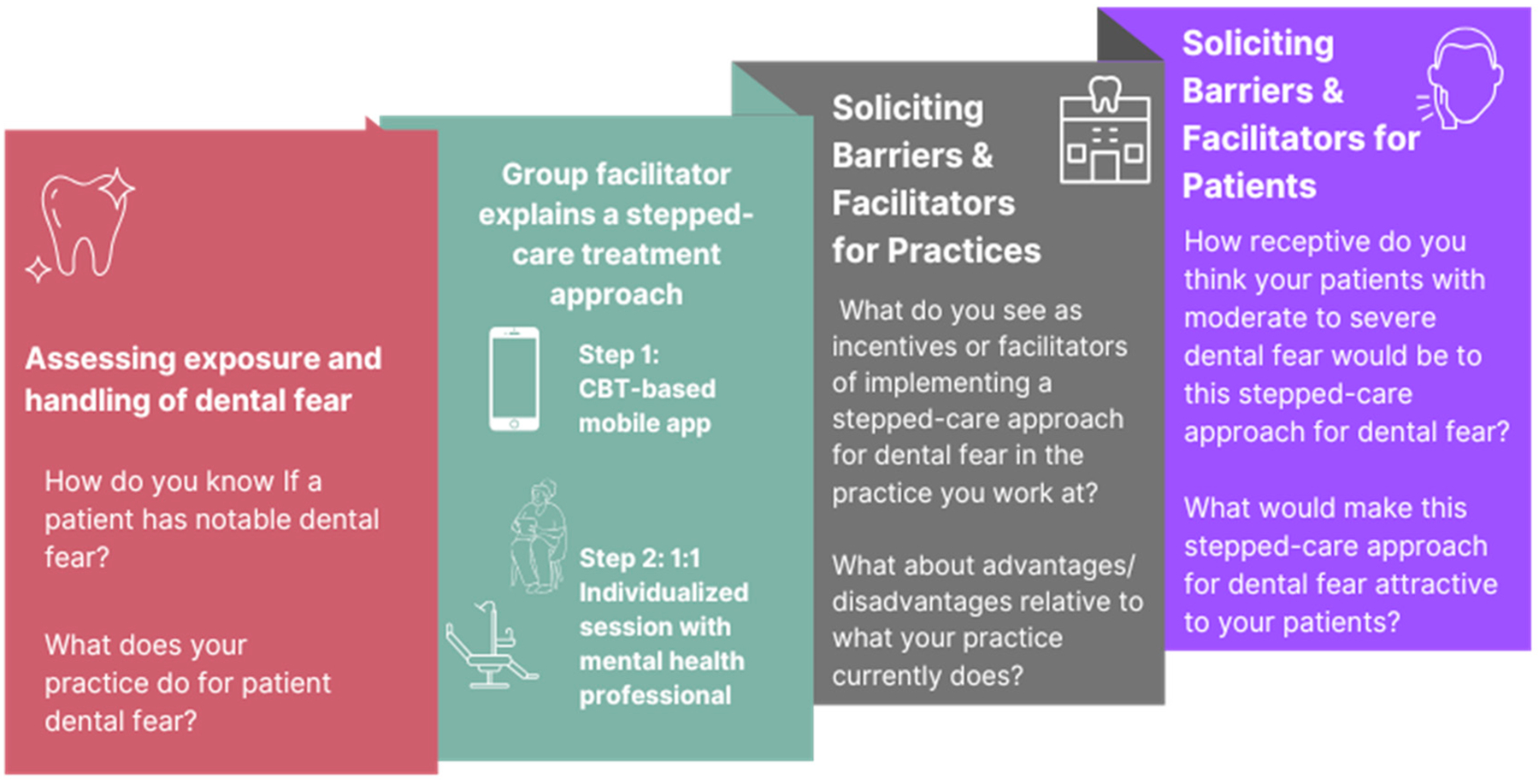
Discussion topic order of focus groups of allied dental professionals.

Follow-up questions probed for the perceived advantages or disadvantages of using the stepped-care approach for treating dental fear and the feasibility of an app-based step. Finally, the facilitators asked participants to provide likely perspectives of dental practitioners and patients at the practices where they had worked. See Figure 1 for the focus group topic sections in order and example questions facilitators asked participants.

The facilitators started discussions by assessing participants’ exposure to dental fear within practices they worked at and then explained our two-step cognitive-behavioral therapy dental fear treatment model. Facilitators then solicited information about perceived receptivity to such a model from the perspectives of dental staff/practices and from patients. Select example questions asked during focus groups are included.

All focus groups were conducted online via the institution’s Health Insurance Portability and Accountability Act (HIPAA)-compliant video conferencing platform and recorded. The audio was uploaded to Otter.ai, which transcribed the sessions. Transcripts were then verified by a research assistant, who listened to the audio recording and modified it for accuracy. All transcripts were de-identified before analysis. In acknowledgment of their time, participants were entered in a raffle to win one of five $50 gift cards. All procedures were approved by the NYU Langone IRB (IRB-FY2020–4535).

### 2.2. Data Analysis

A thematic analysis [29] was conducted. An initial codebook was developed by two research team members and revised with support from a third. The codebook was organized based on the study’s overarching research questions and the focus group protocol. Sections of the codebook focused on (a) how practices identify dental fear in patients, (b) how practices address dental fear, (c) dental practices’ possible reactions to the steppedcare approach, (d) patients’ possible reactions to the stepped-care approach, and (e) clinical factors for adopting the stepped-care approach. The codebook was piloted and revised before being approved by all study team members. A team of five dental students coded the focus group transcripts using Dedoose [30]. A study team member with expertise in mixed-methods research and qualitative data methodology checked all coding to ensure quality and consistency across transcripts.

After coding was completed, conceptually clustered matrices [31] were created to identify themes within and across focus groups. Initial matrices organized the quotes associated with each code and subcode by focus group, with separate matrices for each research question. All quotes associated with a code were carefully read multiple times by two different dental students to identify themes, which were organized in a separate matrix. The themes were then sorted across focus groups and organized to address the study’s research questions.

## 3. Results

### 3.1. Observed Practices for Identifying and Addressing Patients’ Dental Fear

Allied dental health professionals estimated the prevalence of moderate-to-severe dental fear in their practice experience: 0–10% of patients (8.2%), 11–20% (16.3%), 21–30% (26.5%); 31–40% (20.4%), 41–50% (4.1%), 51–60% (16.3%), and 61–70% (8.2%). However, although three-quarters estimated the prevalence to be 21% or above, they also believed dental fear is largely underdiagnosed and not attended to by dentists. Participants reported that, in the practices in which they worked, dentists often did not know that their patients had dental fear or even appeared to ignore overt indicators of fear (e.g., shaking). When participants did observe dentists identifying fear in patients, it was most often due to diagnoses being noted in patients’ charts from previous dental experiences or recognized due to patient behavioral responses during actual procedures. For example, one participant described that in their previous practice “they had behavior readings on the charts. If it was poor behavior, it was likely related to fear”.

Participants identified a wide range of indicators that dental staff can use to determine if a patient may be suffering from dental fear (Table 2). Although these indicators include direct means of communication around dental fear (i.e., verbal indicators, charting), focus-group participants aptly noted that not all patients would directly voice their fear. As a result, they reported dental staff needs to have the ability to recognize signs of fear in other ways, such as universal fear screening, nonverbal indicators (e.g., “posture, their body language kind of shows […] if they’re like a little bit nervous, […] a little bit squirmish, […] their arms crossed together”) or behavioral patterns. Importantly, participants acknowledged that fearful patients often avoid dental care, and as a result, may only present to the dental office when there is an emergency. During one focus group, it was noted that patients with lower socioeconomic status appeared to experience dental fear more often. This observation—empirically demonstrated in previous quantitative research [32]—may be due to these patients not having consistent preventive care and only accessing dental care when their oral health has significantly deteriorated.

The professionals believed that undertreated dental fear creates a burden on dental staff because providing dental care for fearful patients requires different methods than treating a non-fearful patient. In some cases, dentists needed to modify treatment plans to accommodate the patient’s needs, which they felt could lead to compromises in oral healthcare. One example was that patients with dental fear may have difficulty tolerating the dental X-ray sensor, so dentists may have to use panoramic X-rays, which lack detail compared with dental X-rays and are not diagnostic images for finding caries.

Participants observed a wide range of techniques for addressing patients’ dental fears (Table 3). However, across focus groups, the predominant opinion was that, for most patients, either nothing was done to treat dental fear, or the patients were referred for sedation.

Participants offered opinions regarding things that can be done to address patients’ fear in the dental operatory. First, they emphasized reassuring the patient that they are in control of the situation and helping them understand what will be happening throughout the procedure. Second, participants noted the importance of building trust between the patient and dental staff. Third, participants voiced that fearful patients should be scheduled with a familiar dentist. Fourth, the relationships between the patient and other dental staff (e.g., assistants, hygienists, front desk staff) were viewed as critical supports for patients with dental fear. Participants believed hygienists and allied professional staff can often build rapport with patients in ways that dentists may not. In part, these participants suspected that patients associate painful procedures with dentists but not with other staff. Finally, participants suggested that talking to the hygienist about something unrelated may be a useful distraction tool to help patients relax during procedures.

### 3.2. Impressions of the Stepped-Care Approach and Its Feasibility for Treating Dental Fear

Participants’ impressions of the stepped-care approach and its feasibility for treating dental fear reflected factors that would influence its adoption and implementation in dental practices. In discussing these factors, participants anticipated the reactions of both dental staff and patients. Table 4 summarizes the factors identified across focus groups.

Discussions about the adoption of the stepped-care app approach focused on how populations would be impacted long-term. When considering dentists’ perspectives, focus-group participants believed that dentists would be willing to use this method due to its likely impacts on attended (rather than canceled) future appointments with less-fearful patients and improvement in patient–doctor relationships. Similarly, participants believed patients may be willing to engage with the stepped-care approach if they are committed to reducing their dental fear and have access to successful testimonials/data (“If a few people who have used the app and have been able to overcome that fear, advocate for the app, and share their experience with it, and give positive reviews […] it will be easier for people to accept”.). However, “if the fear is so severe, they wouldn’t even think to participate”. They predicted that children and those with special needs may be especially interested.

Participants wondered whether the fearful patient population has access to technology and whether they possess the knowledge and adeptness to use the mobile app itself. If the app were completed at the dental office itself, the focus groups identified several factors that offices would nesed to address for successful implementation, including sufficient auxiliary staff and providing spaces for patients to sit while using the app. Participants also anticipated that dental staff would be concerned about the accessibility and user-friendliness of the app.

All focus groups discussed the time and costs of multi-disciplinary treatment (especially in Step 2). The overarching feeling was that the initial time invested in launching such a coordinated care initiative would likely be high, but decrease over time once the stepped-care approach was integrated with patient care. Cost, however, remained a prevalent concern in both implementation and adoption discussions because treatment costs would potentially increase for both patients and dentists, including (a) lost chair time while a fearful patient is undergoing one-on-one treatment with the mental health provider and (b) patient payments (e.g., if patient had mental health insurance coverage: additional copayments; if patient did not: out-of-pocket expenses for mental health services); and (c) patient lost income (from spending time away from work to complete dental fear stepped-care). As one participant explained, “a lot of dentists, they’re known to just want to just see the patients, get the procedure done, and move on to the next, so I can see time being definitely a barrier”.

Several participants believed that some dentists might be (a) resistant to implementing any procedures that touched on behavioral health (e.g., asking about dental fear, explaining available stepped-care options), (b) lacking in experience addressing patients’ psychological concerns, or (c) unable to see the limitations of their current practices. As one participant explained, “Sometimes dentists, and anybody, they have quite a bit of an ego […] They may believe that they’re perfect in that regard, that nobody can fear them. So [you] have to break through that”. Another added that “some dental professionals might not be comfortable in now playing a more like psychiatric role”. In contrast, participants believed that hygienists would likely play an important role in implementing the stepped-care approach, as they spend more time talking with patients when taking their medical and dental history, and providing regular cleanings and prophylactic treatments.

## 4. Discussion

Overall, study participants believed that a stepped-care approach has promise as an intervention that could meet the needs of patients and dental practices; however, several factors likely need to be addressed for its adoption by practices. Significantly, the experiences of focus group participants corroborated the need for innovation in the management of patient dental fear [3,14]. Although allied dental health professionals had observed a variety of techniques used to manage patient dental fear, the single most frequently witnessed dentist behavior was to do nothing. Consistent with the literature [33–35] participants indicated that dental fear was prevalent, underdiagnosed, and undertreated in the practices in which they had worked. Participants further noted that the dentists they worked with tended only to be aware of fear when patients disclosed, exhibited overt behavioral symptoms, or had pre-existing fear documented in their charts. This is consistent with prior research suggesting that patients’ unpleasant emotions are typically masked in front of dentists [36], and dentists are largely unaware of non-obvious manifestations of fear and anxiety [2] and are poor at assessing patient fear [7]. Thus, relying on dentists alone to manage dental fear is unlikely to mitigate the problem.

Participants additionally mentioned the difficulties patients’ fear can pose to clinic efficiency, echoing empirical research regarding the extra time and financial pressures incurred when treating fearful patients [37]. They also suggested that procedural accommodations made for fearful patients (e.g., using a panoramic X-ray instead of a sensor) may even compromise their care. This was surprising, as minimal intervention dentistry is a frequent recommendation for fearful dental patients [38], who are well known to have worse oral health [2]. Whether accommodations (e.g., the use of more basic procedures) sustain poorer oral health in fearful patients who attend the dentist has not been addressed in the literature. Regardless, focus group discussions supported the supposition that the status quo for handling dental fear is inadequate.

Although overall receptive to the premise of the stepped-care intervention, participants evaluated barriers to implementation from the perceived perspectives of both dental professionals and patients. Cost considerations of the stepped-care approach were thematically similar for both parties and included financial and time costs. Specifically, for dentists, focus-group participants worried about the potential need to hire auxiliary staff, losing chair time to the mental health provider, and how insurance coverage and payment for the mental health provider would work. For patients, participants worried about the need to pay an additional fee for the mental health provider and lost income due to time away from work. This was a particular concern considering the groups’ reporting that they have encountered dental fear more frequently among lower-income adult patients, an observation that has some empirical support [39]. For both parties, participants worried about the time investment—for dental staff to administer and patients to undergo—the intervention. Participants posed that costs would be justifiable for dentists if the stepped-care approach increased clinical efficiency and profit, and for patients, if they were highly motivated to address their fear or part of specific populations that might be particularly inhibited by their dental fear. Overall, discussions of barriers indicated the need to delineate the logistics of adopting a stepped-care approach tailored to individual practices (e.g., insurance or out-of-pocket pay for mental health providers; negotiations between dentist and mental health providers regarding space and resource use; hours during which one-on-one treatment might occur; need for technology support from staff). Given this feedback, a large randomized controlled trial of the stepped-care approach in the U.S. shifted to an entirely virtual approach (i.e., an app for Step 1; one-on-one telehealth treatment session for Step 2; [40]).

Despite the ubiquity of mobile technology (with 85% of American adults owning a smartphone and 88% of individuals’ time using smartphones devoted specifically to using apps; [41]), participants worried about the accessibility of an app-based intervention. In addition to discussing specific features that could make such an app engaging (e.g., pictures, videos), participants expressed concern about individual patients’ technology know-how and the staff burden unfamiliarity could cause. Such concerns emphasize the importance of rigorous iterative usability testing on a diverse sample of fearful dental patients, including elderly individuals, before conducting randomized controlled trials and, eventually, disseminating a mobile app to dental practices [42]. Although this is an important process in the development of any mobile app, it is crucial for the release of mental health apps geared toward self-management of symptoms [43].

In their roles as allied dental health providers, focus group participants were often aware of patient fear that their dentist colleagues failed to notice, corroborating the notion that they have a different type of relationship with patients [43]. Wang et al. (2017) found that fearful patients perceive hygienists and other allied professionals who work with patients (but not front desk staff or non-clinical team staff) as essential to their attendance—communicating accessible information about their treatment and increasing their trust in the process [43].

Furthermore, participants stated that dental hygienists often build rapport with patients in a way dentists do not and affirmed that hygienists would be the likely focal point if a stepped-care intervention for dental fear were to be implemented. Participants emphasized that although dentists might be invested in adopting the intervention to improve their relationships with patients, they may be simultaneously unwilling to engage in the screening, education, and referral process themselves. Across groups, the stepped-care approach resonated with hygienists and other allied dental professionals, who commented that facilitating it aligned with the roles they served in the practices in which they worked. Participants emphasized the importance of buy-in from both dentists and hygienists, as dentists are likely the executive decision-makers for the practices they own, and hygienists are likely to be the individuals screening and referring patients and liaising with mental health partners.

This study’s strengths included a multidisciplinary team (including dentists, dental hygienists, and PhD-level clinical psychologists) that brought differing knowledge, training, and experiences to the study design and research process. In addition, the study specifically focused on hygienists and allied dental professionals, who are uniquely well suited to consider the appeal and drawbacks of any innovation from the perspectives of dentists/practice-owners, other staff, and patients. Indeed, implementation science impels us to engage community providers at various levels to inform the dissemination of any intervention [44].

This study had several limitations. First, the sample was not broadly representative of hygienists and allied professionals in the U.S., having been drawn from self-selected professionals with experience in these fields who were currently enrolled in dental or hygiene school. Second, a stronger evaluation of the intervention’s potential could have been achieved by including a demo of the app prototype or greater detail regarding potential logistics (e.g., requirements for insurance reimbursement) of working in concert with a credentialed mental- health provider. Third, this study was designed to test general receptivity to the concept, and thus needs to be supplemented by future research on the appeal of different possible implementation iterations as a function of practice characteristics. Finally, the need to do a comparable study assessing receptivity to the approach among practicing dentists is self-evident.

Future work should continue to explore receptivity to disseminable models of dental fear treatment among various stakeholders (e.g., dentists, allied dental staff, patients, mental health providers, and insurance company representatives). Evidence-based cognitive behavioral dental fear interventions mediated by technology (mobile app and computerbased protocols, telehealth treatments, and virtual reality programs) hold promise as accessible, cost-effective, potentially efficacious models that can be widely disseminated, without impacting dental practices’ workflows and profitability.

## 5. Conclusions

Participants were unanimous in espousing the importance of improving the management of dental fear and were generally receptive to the idea of a stepped-care approach. The barriers and facilitators they identified provide a roadmap for important pre-adoption steps in refinement and efficacy testing of the treatment (i.e., iterative app usability testing, researching logistical considerations, presenting models for implementation based on practice characteristics, the importance of quantitative and qualitative indicators of efficacy for dental professionals and patients). Given the post-COVID explosion in patient acceptance, and efficacy testing, of mental health treatment via videoconference, the possibilities of dentistry/mental health collaborations that offer little to no negative impact on office workflows are expanding and need to be pursued.

## Figures and Tables

**Table 1.** Participant demographic characteristics.

Demographic Characteristic	M (SD)/% (*n*)
Age (years)	28.5 (5.7)
Experience in hygiene or allied professional role (years)	5.5 (6.1)
Gender Identification	
Female	73.5% (36)
Male	26.5% (13)
Race/Ethnicity	
Asian/Asian-American	32.7% (16)
Black/African-American	2.0% (1)
Hispanic/Latino (of any race)	12.2% (6)
White (Non-Hispanic/Latino)	51.0% (25)
Multiracial	2.0% (1)
Other	8.2% (4)

**Table 2.** Indicators used by dental staff to identify patients’ dental fear.

Verbal Indicators	Behavioral Patterns	Charting	Nonverbal Indicators
Patient tells staff about their dental fear (unsolicited) Patient tells staff about their dental fear in response to screening questions Patient asks many questions Patient has specific requests	Patient avoids dental care Patient only presents for care in emergencies	Fear indicated in patient’s previous medical or dental history	Facial expressions (e.g., grimacing, flinching) Body language (e.g., crossed arms, clenching chair, sweating) Paraverbal signs (tone, volume)

**Table 3.** Observed techniques for addressing patients’ dental fear.

Distraction	Relaxation	Treatment Planning	Medications	Scheduling
Distract (*conversation*) Talking to a hygienist instead of a dentist TV/music Toys	Iatrosedation^49^ (*deliberate changes in behavior and communication to calm patients*) Establish routine	Step-by-step explanation Desensitization Patients raise hand for pain Modify treatment plan (*for easier procedures*)	Nitrous IV sedation General anesthesia	Shorter procedure Longer appointments Familiar clinician

**Table 4.** Factors perceived to influence the adoption and implementation of the stepped-care approach.

	Dental Staff’s Response	Patients’ Response
Adoption Factors	Belief that it will improve patients’ perception of dentist/practice Smaller/private offices Cost consideration Must increase clinical efficiency and profit	Population Moderate-severe fear Younger/pediatric Special needs Patient motivation to treat fear Efficacy data or testimonials
Implementation Needs	Auxiliary staff for administration Access and knowledge of how to use technology amongst patient population Security and liability of app information Space and time required in office Time of app administration Features of app (e.g., pictures, multiple languages, videos) Cost consideration Chair time Insurance coverage Cost of auxiliary staff Increased appointment time May require multiple sessions	Access and knowledge of how to use technology in patient population Cost consideration Additional service fee Additional time would require patient to spend more time away from work, etc.

## Data Availability

This qualitative data were extracted from transcribed focus group interviews with allied dental professionals for the purposes of this study’s aims. Full focus group session transcriptions, which contain some potentially identifying information, will not be shared with any data depositories to protect participants’ privacy. Should any researchers be interested in redacted transcripts of the focus groups for the purpose of additional analyses, they can email a request to kelly.daly@nyu.edu.
